# Supplementary material for: Identification of the master sex determining gene in Northern pike (Esox lucius) reveals restricted sex chromosome differentiation
Source: PLoS Genet. 2019 Aug 22;15(8):e1008013. doi: 10.1371/journal.pgen.1008013 (PMC6726246; doi:10.1371/journal.pgen.1008013)
Supplement: S1 File — (DOCX) [file pgen.1008013.s001.docx]

# Supplementary results

**Pool-seq analysis revealed ~180 kb of Y-specific sequences**

Three Nanopore genome contigs (tig00003316, tig00003988 and tig00009868) with male-specific coverage were identified in *E. lucius* based on pool-seq analysis results.

Contig tig00003316 from the Nanopore genome sequence, containing *amhby,* has three repeated regions (shown in **Figure S4A**), and its end is homologous to a region of scaffold 1067 from the reference XX genome sequence (megablast, e-value = 0, identity = 96%). These three repeated regions and the end of the contig had both male and female coverage in the pool-seq, while in the other 73 kb of the contig, females had a coverage of 0.54 ± 1.4 with 81% of the bases with no mapped female reads, and males had a coverage of 14 ± 5.6 (0.44x genome average). The sex-specific coverage pattern on this contig indicates that it is totally absent from the X chromosome, and thus almost entirely belongs to the inserted male determining region on the Y chromosome.

Contig tig00003988 contains a repeated ~5 kb region at the proximal end **(**annotated on **Figure S4B)**. Apart from this repeated region, the rest 30 kb of this contig exhibits a coverage of 0 in females for 97% of all bases, while males have a coverage of 12 ± 4.0 (0.38x genome average).

Contig tig00009868 contains a repeated region near the center of the contig (annotated on **Figure S4C**). The proximal ~70 kb of this contig showed homology to a region on LG24 spanning from ~0.72Mb to ~0.80 Mb (megablast, e-value = 0, identity = 95%) and did not contain any male-specific coverage windows. The other 66 kb of tig00009868 had a male coverage of 15 ± 4.9 (0.47x genome average) and a female coverage of 1.8 ± 1.8. The low but non-zero female coverage suggests that half of tig00009868 is located in a region with reduced recombination between the X and Y chromosomes and falls in the regions enriched with MSS on LG24.

The repeat content of these three contigs was compared to that of the entire Nanopore genome assembly (**Table S6**). The Nanopore genome assembly contains 2.8% of repetitive sequences and these three contigs contain a slightly higher amount of repetitive sequences at 3.98%. While these three contigs contain higher amount of all different categories of repetitive elements than the entire Nanopore genome assembly, the most pronounced difference is found in the class of simple repeats, which is 2.95% in these three contigs and 1.96% in the entire Nanopore genome assembly.

**Estimation of divergence time between *amha* and *amhby* suggests an ancient duplication event**

The lineage-specific median Ks is estimated to be 0.434 between *E. lucius* and *Salmo salar* [1] based on full-length cDNA sequence. Furthermore, the divergence time between *E. lucius* and *Salmo salar* is estimated to be between 100 and 130 My [2,3]. With this information, we could estimate a mean lineage specific divergence rate for the *E. lucius* lineage to be between 0.0033 and 0.0043 change/site/My.

The Ks value between *amha* and *amhby* of *E. lucius* is calculated to be 0.328 using the KaKs_Calculator 2.0 [4] with the NG method and standard model. Assuming that *amha* and *amhby* evolved independently at a rate equal to the lineage-specific rate of divergence [5], the combined divergence rate would then be between 0.0066 and 0.0086 change/site/My. We then estimated a rough divergence time between *amha* and *amhby* to be between 38 and 50 million years. In addition, we obtained a Ka value of 0.174 between *amha* and *amhby* of *E. lucius* and a Ka/Ks value of 0.531.

## References

1. Leong JS, Jantzen SG, von Schalburg KR, Cooper GA, Messmer AM, Liao NY, et al. Salmo salar and Esox lucius full-length cDNA sequences reveal changes in evolutionary pressures on a post-tetraploidization genome. BMC Genomics. 2010;11: 279. doi:10.1186/1471-2164-11-279

2. Betancur-R R, Broughton RE, Wiley EO, Carpenter K, López JA, Li C, et al. The tree of life and a new classification of bony fishes. PLoS Curr. 2013;5. doi:10.1371/currents.tol.53ba26640df0ccaee75bb165c8c26288

3. Near TJ, Eytan RI, Dornburg A, Kuhn KL, Moore JA, Davis MP, et al. Resolution of ray-finned fish phylogeny and timing of diversification. Proc Natl Acad Sci USA. 2012;109: 13698–13703. doi:10.1073/pnas.1206625109

4. Wang D, Zhang Y, Zhang Z, Zhu J, Yu J. KaKs_Calculator 2.0: A Toolkit Incorporating Gamma-Series Methods and Sliding Window Strategies. Genomics Proteomics Bioinformatics. 2010;8: 77–80. doi:10.1016/S1672-0229(10)60008-3

5. Chen S, Zhang G, Shao C, Huang Q, Liu G, Zhang P, et al. Whole-genome sequence of a flatfish provides insights into ZW sex chromosome evolution and adaptation to a benthic lifestyle. Nature Genetics. 2014;46: 253–260. doi:10.1038/ng.2890
