# Supplementary figures and images for: Identification of the master sex determining gene in Northern pike (Esox lucius) reveals restricted sex chromosome differentiation
Source: PLoS Genet. 2019 Aug 22;15(8):e1008013. doi: 10.1371/journal.pgen.1008013 (PMC6726246; doi:10.1371/journal.pgen.1008013)

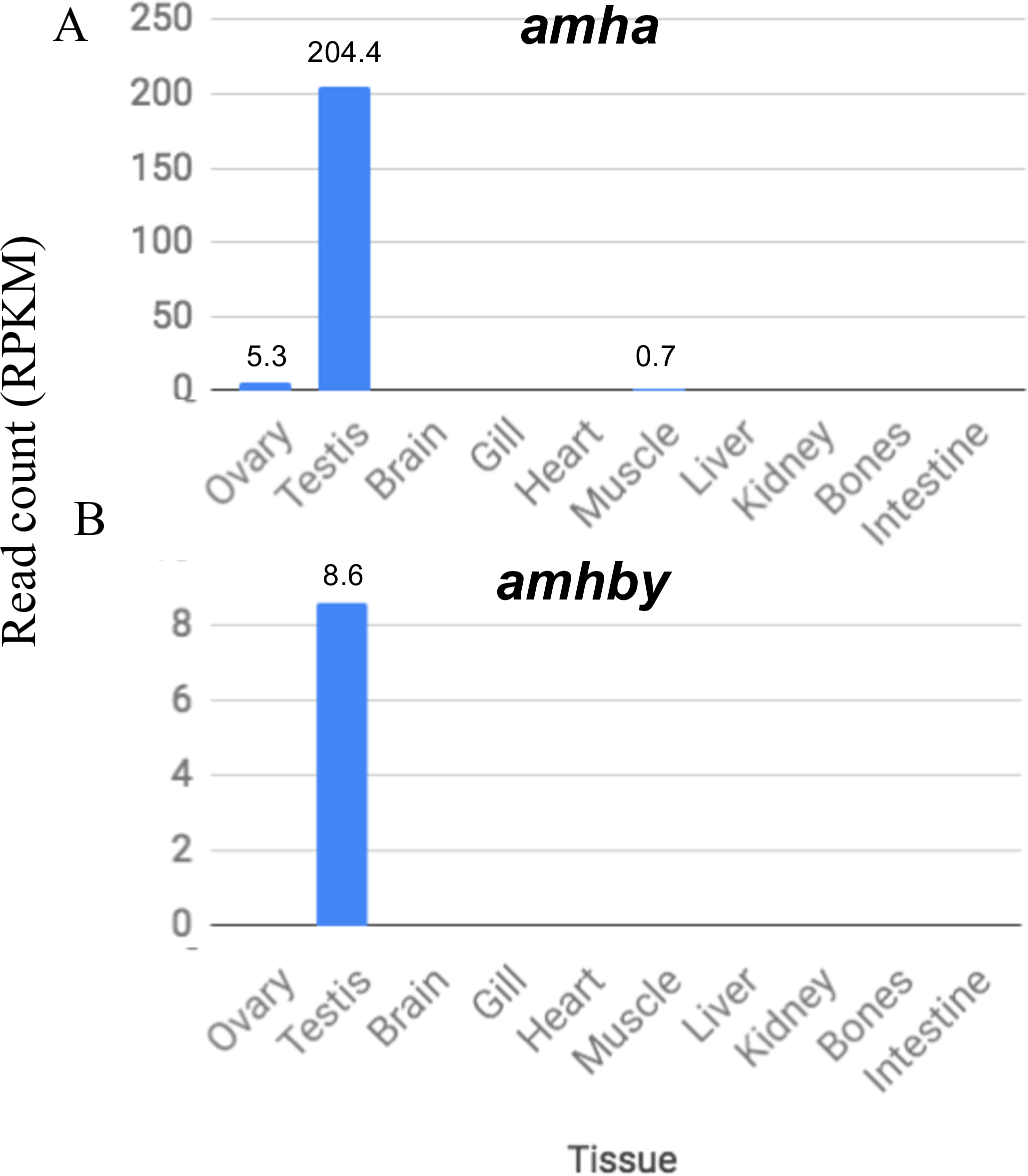

Supplement: S1 Fig — Data was obtained from the Phylofish database (http://phylofish.sigenae.org/) and was normalized by reads per kilobase of sequence per million reads for each transcript in each library for amha (A) and amhby (B). Non-zero values are indicated on top of the bar. (TIF) [file pgen.1008013.s002.tif]

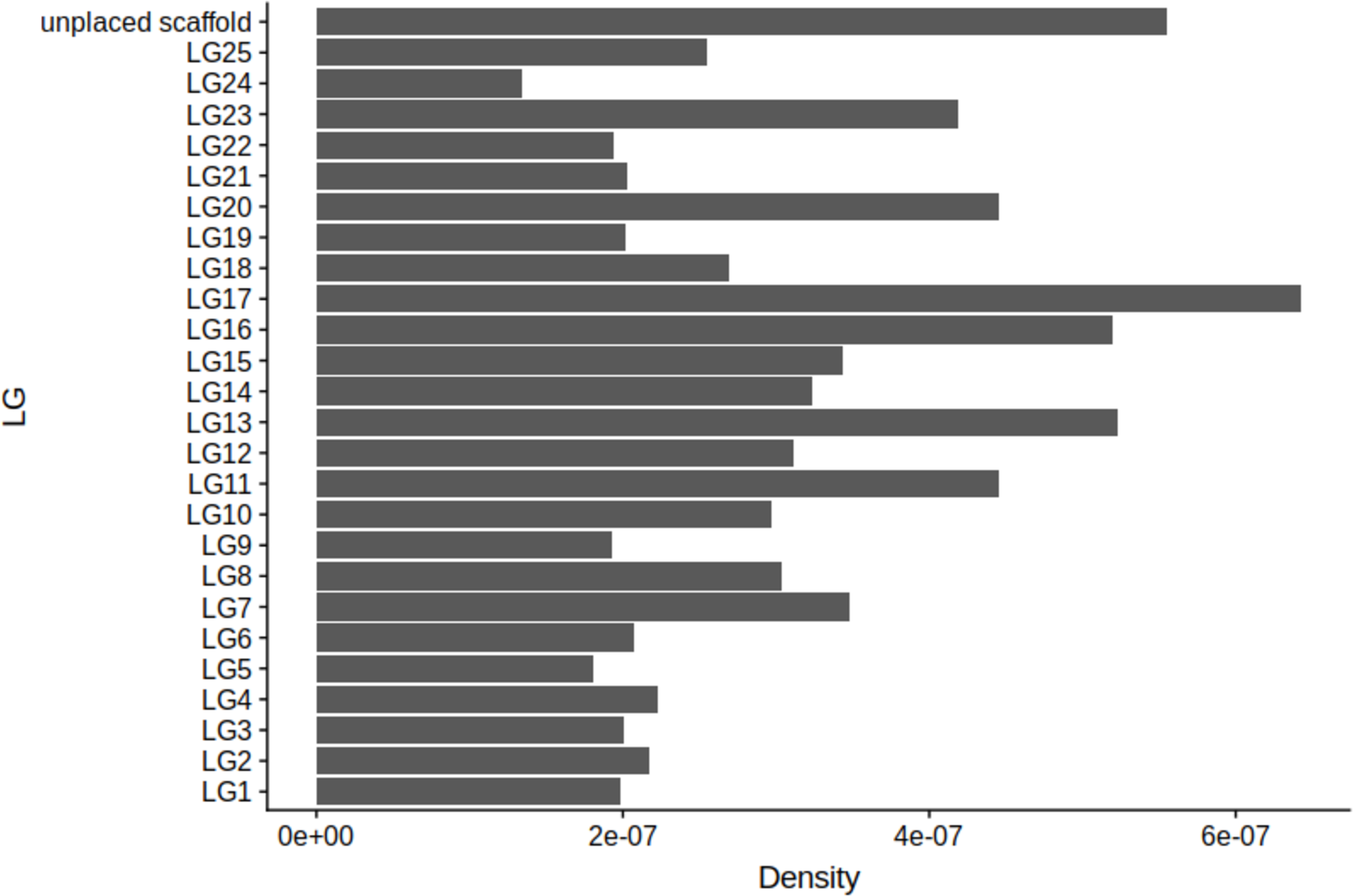

Supplement: S2 Fig — Total number of transposable element domain HTH_Tnp_Tc3_2 divided by the length of each chromosome was plotted for each of the 25 LGs and unplaced scaffolds in the female reference genome (GenBank assembly accession: GCA_004634155.1). amha is located on LG 08 and amhby is located on LG 24. (TIF) [file pgen.1008013.s003.tif]

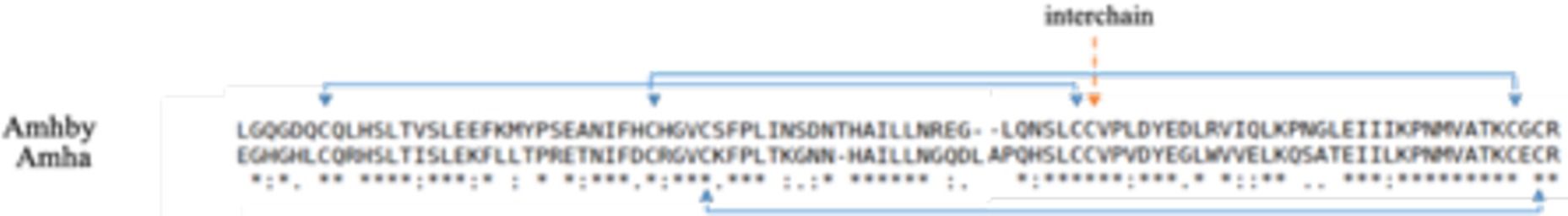

Supplement: S3 Fig — The seven conserved cysteines are highlighted by colored triangles: blue triangles for cysteines involved in forming intra-chain disulfide bonds and a red triangle for the cysteine involved in forming inter chain disulfide bonds. (TIF) [file pgen.1008013.s004.tif]

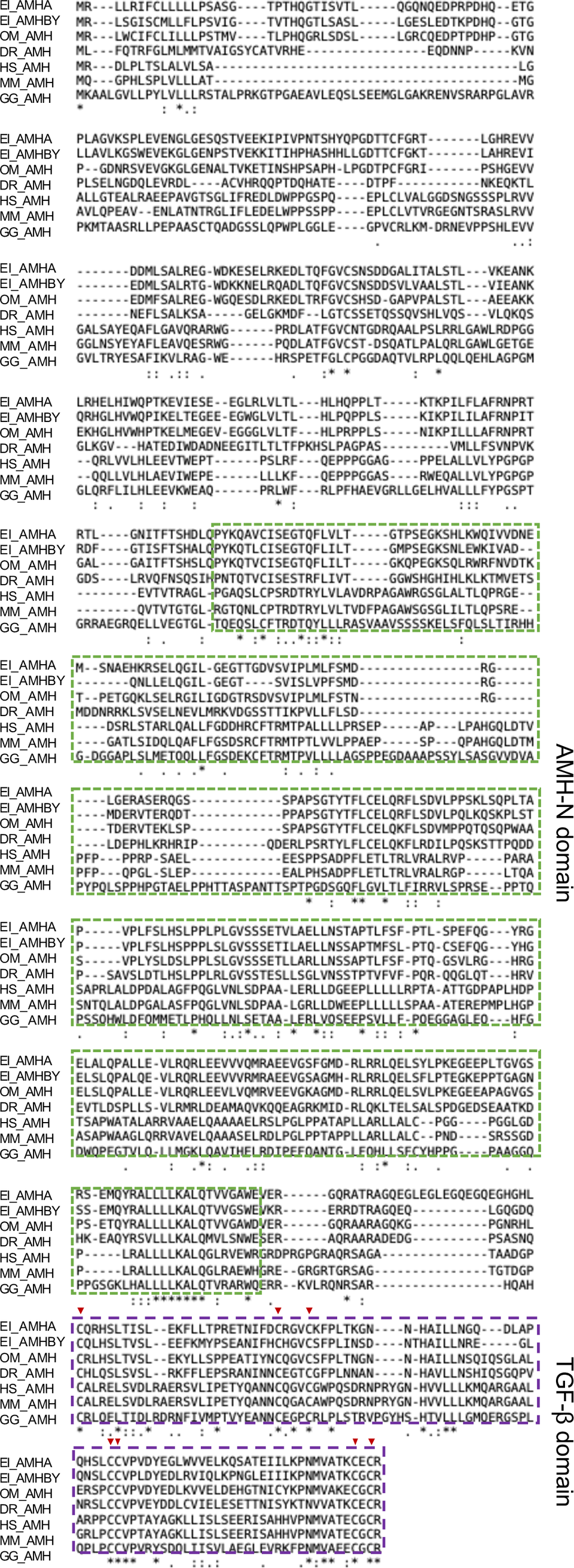

Supplement: S4 Fig — The AMH domain at the N-terminus is highlighted with green dashed-line boxes, and the TGF-β domain at the C-terminus is highlighted with purple dashed-line boxes with the seven conserved cysteines indicated by the red triangles. EL: Esox lucius, XP_010870000.1(Amha) MK355503 (Amhby); OM: Oncorhynchus mykiss, XP_021459508.1; DR: Danio rerio, NP_001007780.1; HS: Homo sapiens, AAH49194.1; MM: Mus musculus, NP_031471.2; GG: Gallus gallus, NP_990361.1. (TIF) [file pgen.1008013.s005.tif]

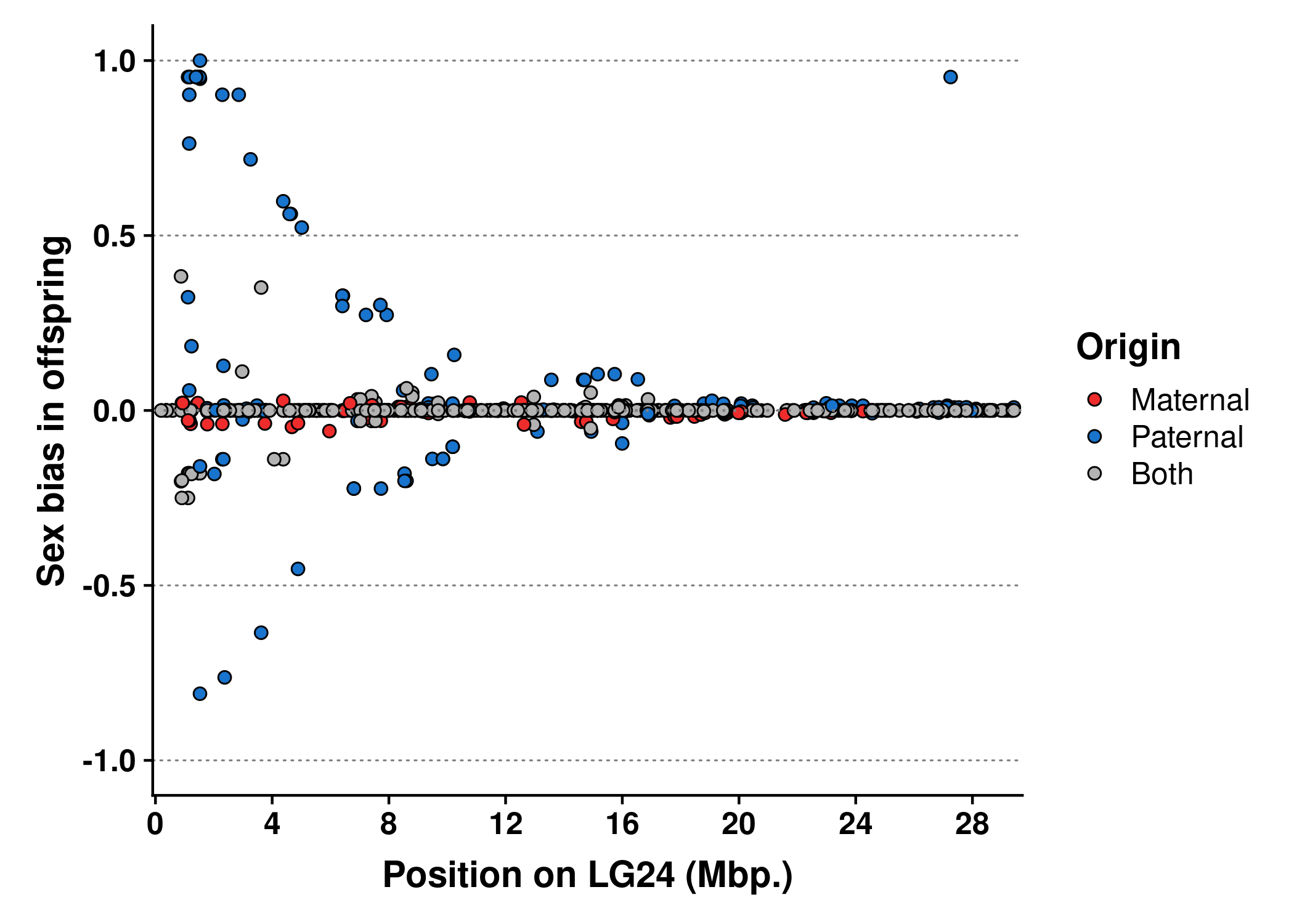

Supplement: S5 Fig — Sex bias in offspring, defined as the difference between the proportion of male offspring and the proportion of female offspring in which a marker is present, is plotted against genomic position of LG24 for all RADSex markers aligned to this chromosome. Markers found in the dam but not in the sire (i.e. maternal-specific markers) are colored in red, markers found in the sire but not in the dam (i.e. paternal-specific markers) are colored in blue, and markers found in both the sire and the dam are colored in grey. Maternal-specific markers and markers found in both parents were evenly distributed among male and female offspring along the entire chromosome. However, paternal markers aligned to the first one third of the chromosome segregated in either male or female offspring. This sex bias in marker segregation is strongest at the proximal end of the chromosome and decreased gradually along the chromosome, corelating with a marker’s physical distance to the sex locus. (TIF) [file pgen.1008013.s006.tif]

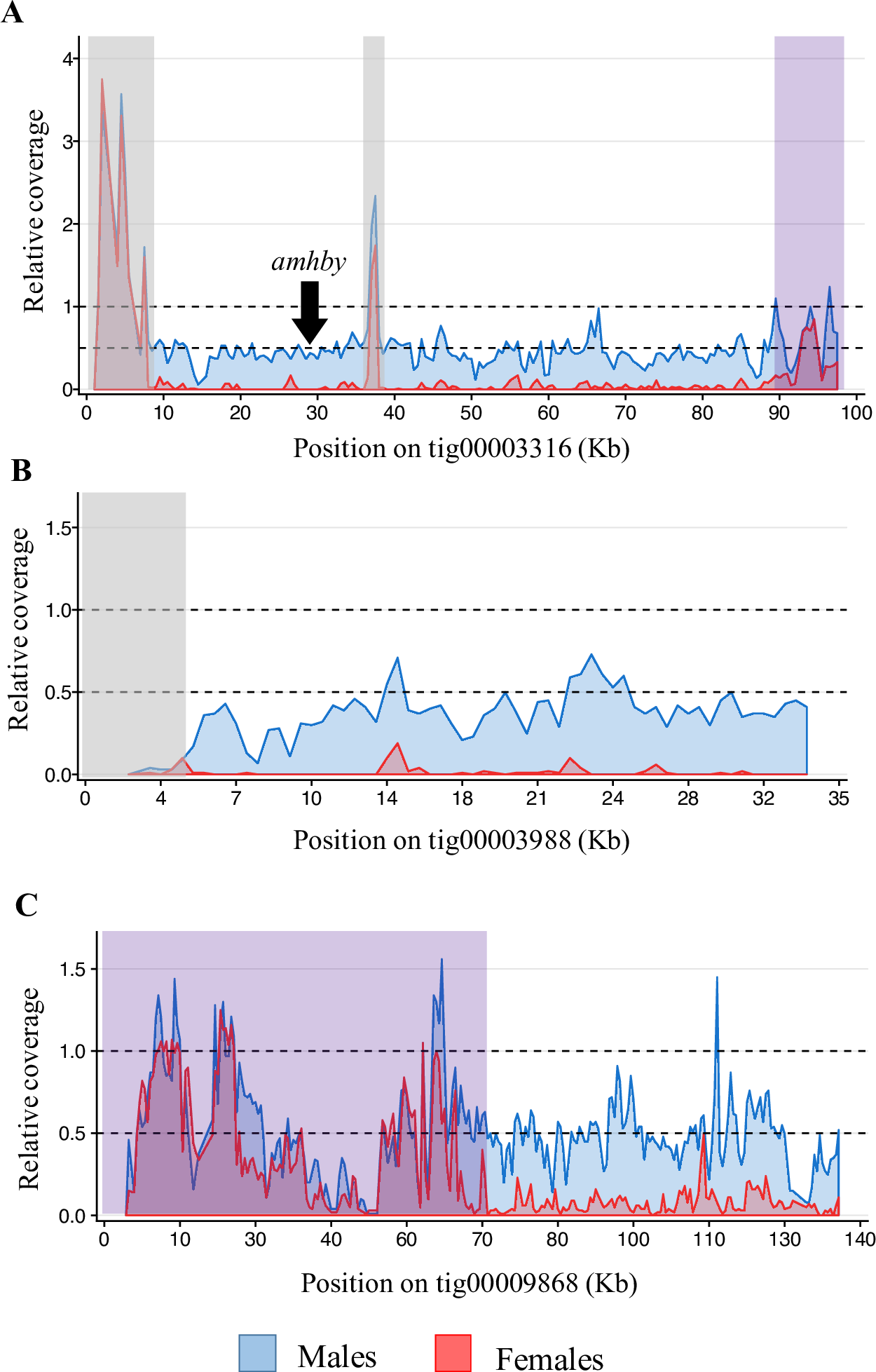

Supplement: S6 Fig — A-C: Three contigs containing regions with only male coverage in the Oxford Nanopore assembly. Relative coverage of male and female reads are indicated by blue and red lines, respectively. The location of amhby on tig00003316 is indicated by a solid black arrow. The lower dotted line indicates 0.5 genome average coverage and the higher dotted line indicates genome average coverage. The grey shaded regions correspond to repeated elements and the purple shaded regions correspond to regions with strong homology with the reference genome (GenBank assembly accession: GCA_000721915.3) scaffold1067. (TIF) [file pgen.1008013.s007.tif]

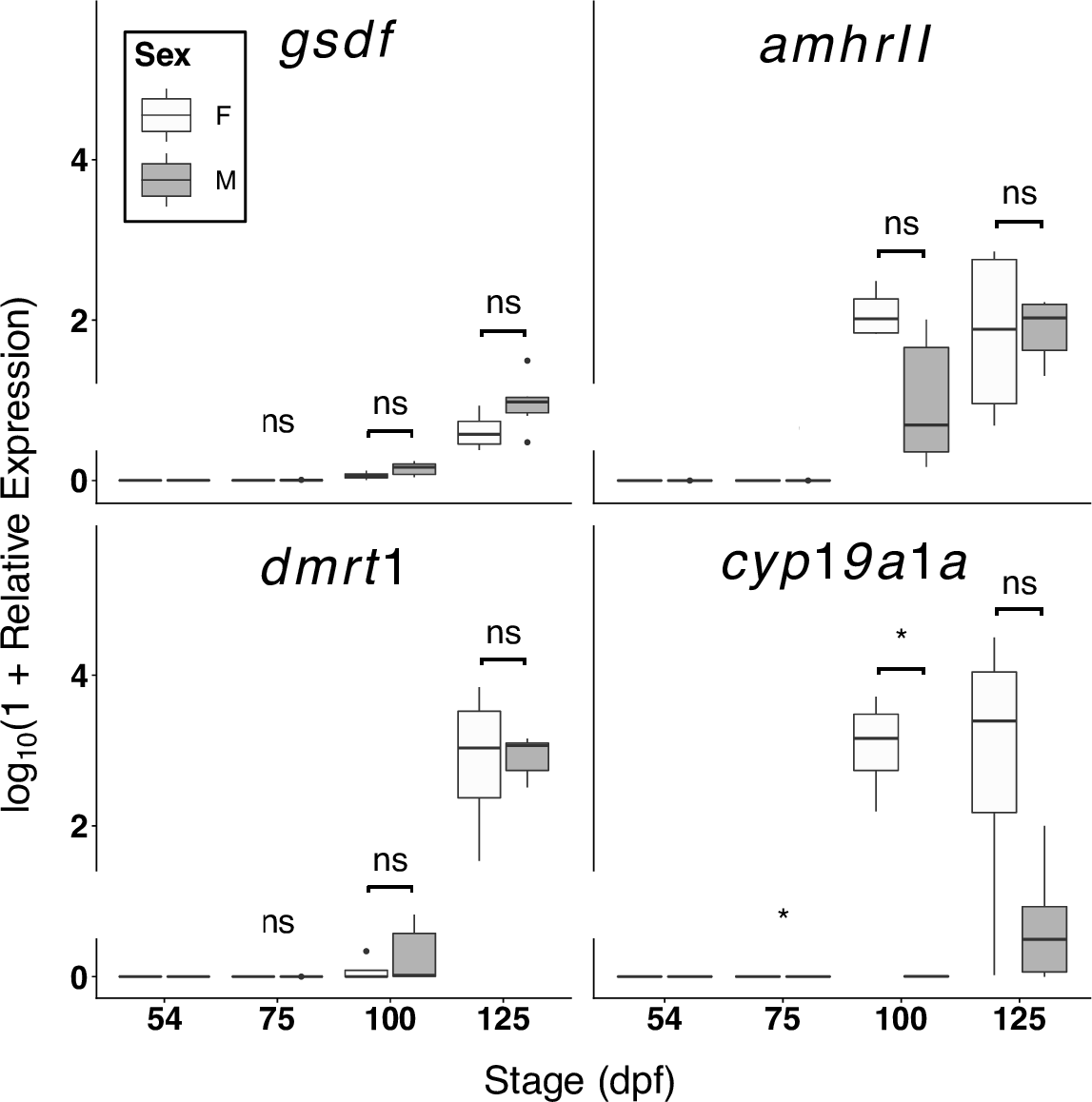

Supplement: S7 Fig — Boxplots showing the first quantile, median, and the third quantile of gsdf, dmrt1, cyp19a1a and amhrII mRNA expression. Outliers are displayed as dots. The log10 of the expression of mRNA of these four genes were measured with qPCR at 54, 75, 100, and 125 days post fertilization in male and female trunks of E. lucius. Significance levels of p-value are given for Wilcoxon signed rank test between male and female expression at each time point: * P ≤ 0.05, ** P ≤ 0.01, *** P ≤ 0.001 and ‘ns’ indicates P > 0.05. (TIF) [file pgen.1008013.s008.tif]

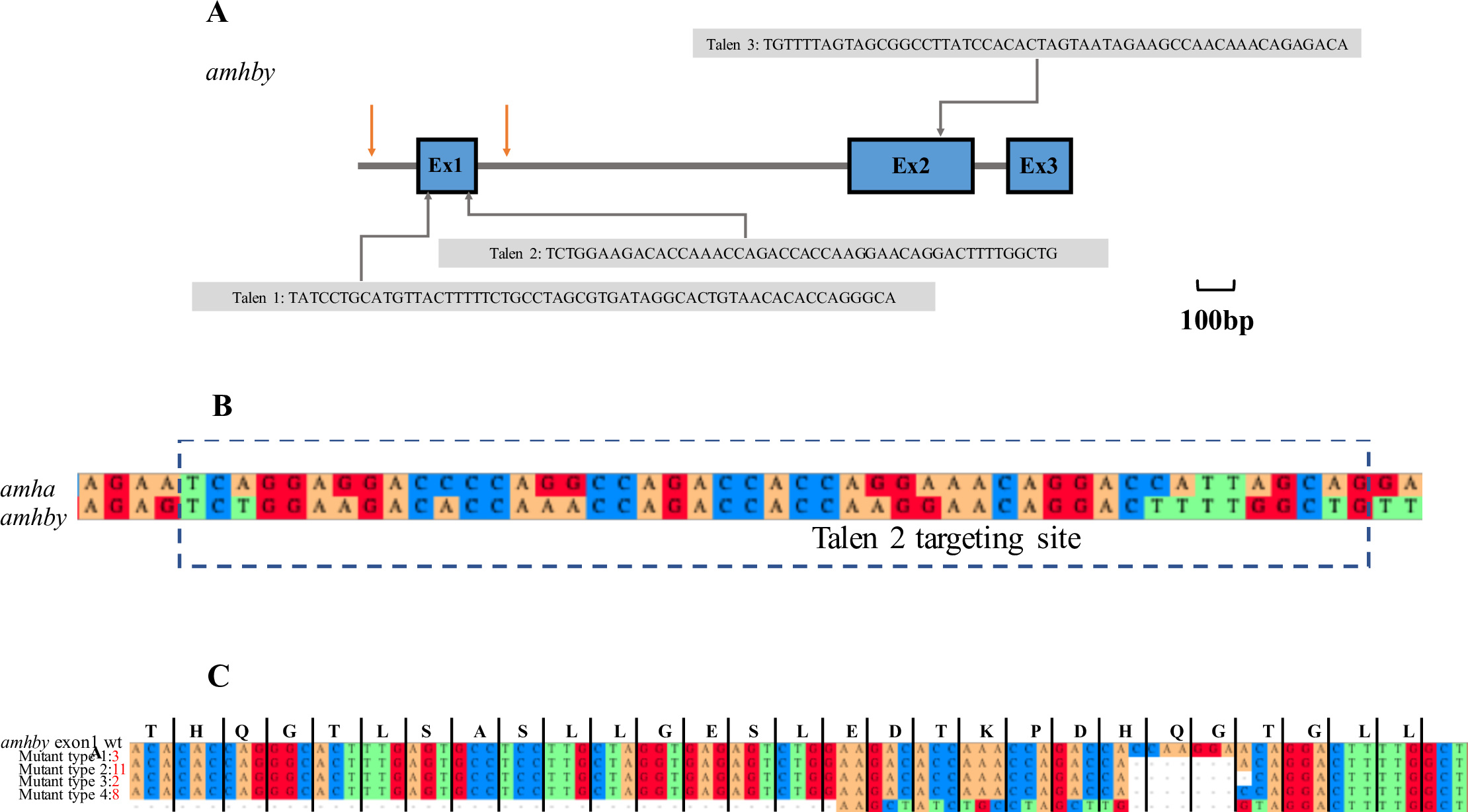

Supplement: S8 Fig — A: Design of TALENs targeting amhby exon 1 and exon 2. Grey arrows indicate the position targeted by TALENs with the corresponding sequences in grey boxes. Orange arrows indicate the position of primers used for genotyping of amhby knockout mutants. B: Alignment of amha and amhby sequence around the region targeted by Talen 2 designed specifically to cleave amhby sequence. Talen 2 targeting sequence is highlighted by a black dashed line box. C: Alignment of amhby exon 1 sequences between the wildtype and three different mutants showing different sequences deletion. The number of G1 animals with each type of the mutation is indicated by the red number. (TIF) [file pgen.1008013.s009.tif]

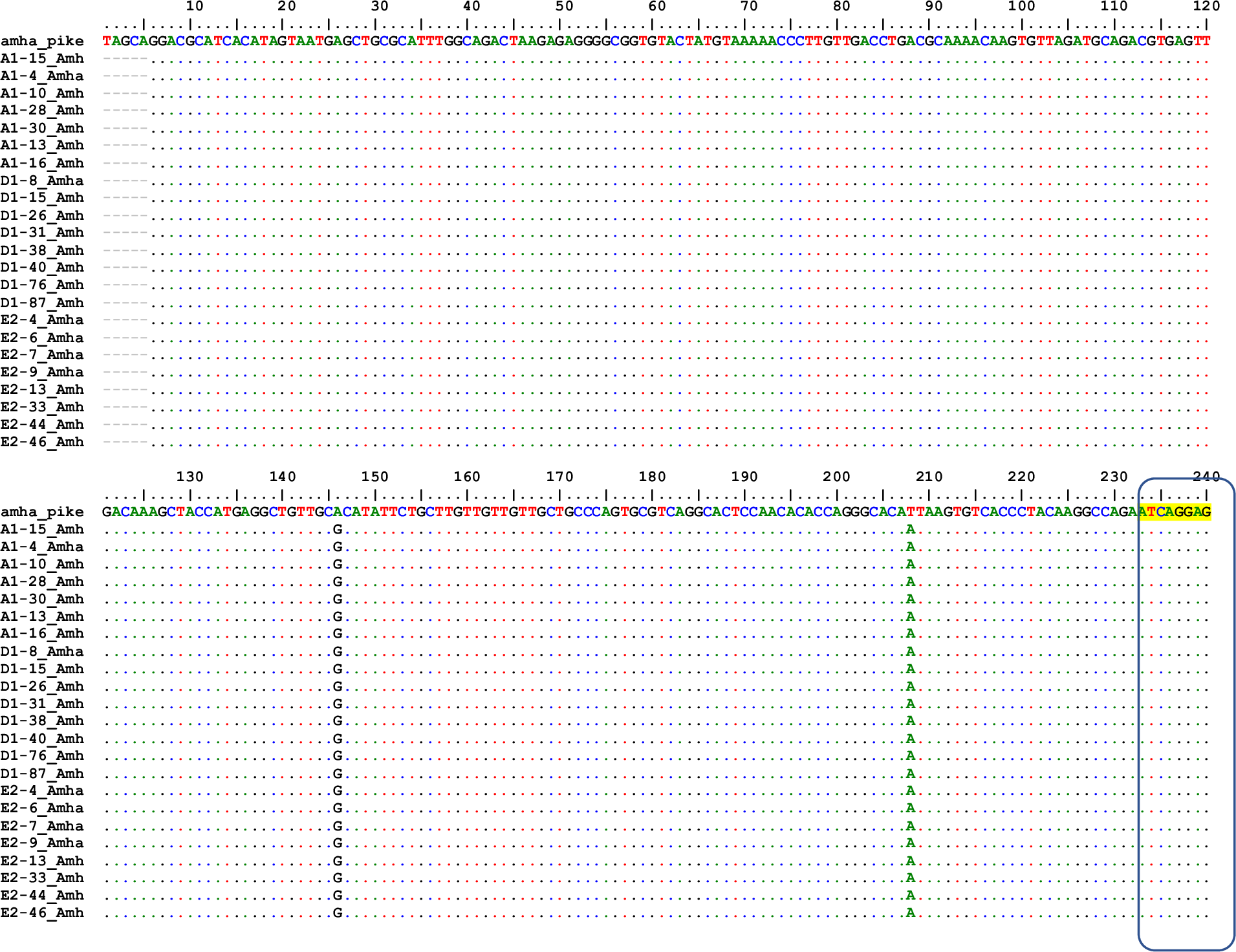

Supplement: S9 Fig — Identical bases to the reference are represented as dots and the regions targets by TALENs on amhby are highlighted in yellow in the reference sequence. (TIF) [file pgen.1008013.s010.tif]
